# Effect of Purple Corn Anthocyanin on Antioxidant Activity, Volatile Compound and Sensory Property in Milk During Storage and Light Prevention

**DOI:** 10.3389/fnut.2022.862689

**Published:** 2022-03-23

**Authors:** Xing-Zhou Tian, Xu Wang, Chao Ban, Qing-Yuan Luo, Jia-Xuan Li, Qi Lu

**Affiliations:** Key Laboratory of Animal Genetics, Breeding and Reproduction in the Plateau Mountainous Region, Ministry of Education, College of Animal Science, Guizhou University, Guiyang, China

**Keywords:** anthocyanin, antioxidant activity, volatile compound, color measurement, sensory evaluation

## Abstract

The aim of this study was to observe the effect of purple corn anthocyanin on the light-induced antioxidant activity, free radicals, volatile compounds, color parameters, and sensory properties of milk during storage. There were four groups: (1) negative control, no addition of anthocyanins + exposure to fluorescent light (NC); (2) positive control 1, no addition of anthocyanins + protected from fluorescent light (PC1); (3) positive control 2, the addition of 0.3% (w/v) anthocyanins + exposure to fluorescent light (PC2); and (4) the addition of 0.3% anthocyanins + protected from fluorescent light (AC). The results indicated that the concentration of antioxidant activity parameters in the NC group decreased during the entire storage period, whereas antioxidant activity parameters were unchanged except for the glutathione peroxidase (GSH-Px) in the AC group. Moreover, the NC group showed lower levels of 2,2-diphenyl-1-picrylhydrazyl (DPPH) scavenging activity and higher levels of superoxide anion and hydrogen peroxide than the other groups after 1 d of storage period. The extent of malondialdehyde accumulation and lipid peroxidation in the control groups were greater than that of the AC group. Twenty-two volatile compounds were determined in milk, which consisted of eight alcohols, three ketones, five aldehydes, two esters, and four hydrocarbons by headspace gas chromatography mass spectrometer analysis. Specifically, individual aldehydes, esters and hydrocarbons in the AC group remained at relatively stable values during storage relative to the other three groups. Stronger positive correlations were detected between several antioxidant activities (superoxide dismutase, GSH-Px) and DPPH scavenging activity as well as total ketones in milk. Adding of anthocyanin did not impact on the color values of L^*^, a^*^ and b^*^ in light-protected milk during the entire storage period. Some sensory evaluation parameters (flat, garlic/onion/weedy, oxidized-light, oxidized-metal, rancid) in AC group were significantly higher than that of the control group at the end of the period. In conclusion, the current study revealed that the addition of purple corn anthocyanin pigment to light-protected milk had the potential to prevent lipid oxidation, enhance antioxidant activity, maintain volatile compounds and increase the sensory scores.

## Introduction

Milk is a wholesome, natural food with high nutrition and easy absorption and is popular among people. The unsaturated fatty acids (UFAs) in milk could strengthen the human immune system, playing important roles in body health (1). However, milk is prone to oxidation influenced by promoting oxidation components (such as flavor, metal ions, and oxidative enzymes) during the storage period (2). Indeed, lipid oxidation has been a major contributor to milk flavor defects, and oxygen (O_2_), metal ions, heat, and light are the main factors that affect lipid oxidation in the milk system because they contribute to the formation of several lipid radicals, lipid hydroperoxides, and volatile compounds (3).

Specifically, volatile compounds play an important role in the acceptability and quality of milk. Studying volatile components would help to better understand the change of milk. The volatile compounds in cow milk was analyzed by Toso et al. (4), and forty-one volatile compounds were identified, including eight ketones, nine aldehydes, eight alcohols, six hydrocarbons, three sulfur compounds, four esters, and three terpenes. Many volatile compounds, such as alcohols, esters, and hydrocarbons, are produced by the oxidation of UFAs in milk (5). There were two pathways for oxidation in milk: (1) dependent upon the superoxide radical, which can be generated by enzymes such as xanthine oxidase and lactoperoxidase; and (2) dependent upon hydroxyl radical and added copper (6). Hence, the antioxidant activity of milk has practical value for the food industry, especially for food formulations. Milk antioxidants can function by removing the formation of radicals or scavenging FR to prevent milk oxidation, improving milk antioxidant activity (7). In addition, natural antioxidants are usually added to milk as FR scavengers. For example, Pihlanto (8) demonstrated that natural antioxidants could enhance antioxidant activity and prevent the oxidation reaction in milk.

The natural active polyphenol compounds are responsible for the total antioxidant potential of many fruits and other purple materials (9). Anthocyanins are a source of secondary metabolites of plants that are effective natural antioxidant and free radical (FR) scavengers, have various kinds of significant physiological functions for consumers, and have broad prospects for development and application (10). Therefore, anthocyanins can improve the activities of antioxidant enzymes, such as superoxide dismutase (SOD), glutathione peroxidase (GSH-Px), and catalase (CAT), to further inhibit FR in milk. Indeed, anthocyanins have been reported to show strong antioxidant potential to protect against lipid oxidation in milk during the storage period (11). Silva et al. (12) reported that anthocyanin-rich Isabel grapes positively influenced the quality characteristics of goat milk yogurt during refrigerated storage.

Milk was suggested to reduce oral bioavailability of anthocyanins, whereas it could be absorbed by humans to improve people's health. However, milk is susceptible to light-induced oxidation because of the photosensitizer riboflavin and lipids, and they are the primary targets for photooxidation. Light-induced oxidation results in lipids quickly producing FR or the very reactive singlet O_2_, affecting the sensorial product quality and degrading valuable nutrients into oxidation products (13). We hypothesized that the addition of purple corn anthocyanin pigment (PCP) could prevent lipid oxidation, improving the antioxidant activity of light-protected milk during the storage period. In this regard, to provide a deep understanding of anthocyanins as radical scavengers for milk, the current study investigates the effect of anthocyanin pigments of purple corn on antioxidant activity, oxidative stability, volatile compounds, color parameters and sensory evaluation in milk during storage and light prevention.

## Materials and Methods

### Sample Collection

The raw cow milk samples were collected from Guizhou University Farm (Guiyang, China). The milk was immediately mixed together, placed into a plastic bucket with an ice pack and transported to the laboratory. The addition of anthocyanin levels and methods were performed according to Güneşer (14) and Serafini et al. (15) with minor modifications. Briefly, all milk samples were prepared by the addition of PCP (0 and 0.3%, respectively) and blended vigorously with a blender (TMHL-200CL, Tianjin Taist Instrument Co., Ltd., Tianjin, China) for 30 s. In addition, high pasteurization (75°C for 15 s) could ensure milk hygienization while producing smaller losses in antioxidant potential (16) and could maintain the PCP concentration (17). Hence, milk samples were pasteurized at 75°C for 15 s using a minipasteurizer (Shandong Zolanbo Electrical Equipment Co., Ltd., Shandong, China). The four groups were as follows: (1) negative control, no addition of anthocyanins + exposure to fluorescent light (NC); (2) positive control 1, no addition of anthocyanins + protected from fluorescent light (PC1); (3) positive control 2, the addition of 0.3% (w/v) anthocyanins + exposure to fluorescent light (PC2); and (4) the addition of 0.3% anthocyanins + protected from fluorescent light (AC). The NC and PC1 groups were placed under fluorescent light bulbs (20 cm; 23 W, E27 crew-type, Philips, Amsterdam, the Netherlands), and the PC2 and AC groups were placed under the same conditions, but they were obtained by covering sample bottles with aluminum foil. Light exposure was regulated at an intensity of 1,100 to 1,300 lx throughout the experimental period (18). All samples were placed into 150-mL plastic tubes (Nanjing Metasequoia Technology Co., Ltd., Nanjing, China) within 2 h and kept at 4°C cooler for periods of 0, 1, 3, and 7 d, respectively. There were six replicates of each sampling time point. Milk has been properly stored by freezing at −80°C until the analysis of antioxidant activity, free radicals, volatile compounds, color measurement and sensory evaluations were conducted according to the reference of Smith et al. (19).

### Anthocyanin Composition

The PCP material was purchased from Nanjing Herd Source Biotechnology Co., Ltd., Nanjing, China. The anthocyanin composition of PCP was determined by High performance liquid chromatography-mass spectrometer (MS)/MS according to the method of Tian et al. (10). The PCP had 2,619 μg/g total anthocyanins.

### Milk Composition

Fresh milk was collected and mixed thoroughly, and the pH value of milk was detected immediately using a portable pH meter (pH 818, smart sensor, Guangdong, China). The pH meter was calibrated using standard pH solutions (pH values of 4.0, 6.8, and 9.18) before measurement. Dry matter (DM) was determined by the method of Güneşer (14). The milk compositions of protein, fat, lactose, total solids (TS), and solids-not-fat (SNF) were measured by a MilkoScan analyzer (MilkoScanTM FT2, FOSS, Hillerod, Denmark). The results were as followed: pH was 6.52, DM was 12.24%, protein was 3.21%, fat was 3.42%, lactose was 4.62%, TS was 12.74%, and SNF was 9.32%.

### Antioxidant Activity, Free Radical, and Lipid Peroxidation Parameters

The milk sample was thawed and centrifuged at 10,000 × g for 30 min at 4°C (Gallop Technology Co., Ltd., Dongguan, Guangdong, China) and the supernatant was collected and mixed with 4% acetic acid before centrifugation at 10,000 × g for 30 min at 4°C. The supernatant was immediately transferred to a 1.5-mL tube and stored at −80°C until further analysis for antioxidant activity, free radical, and lipid peroxidation parameters (20). The antioxidant activity parameters of total antioxidant capacity (TAC), SOD, GSH-Px, oxidized glutathione (GSSG), reduced glutathione (GSH), and CAT; FR parameters of superoxide anion (${\text{O}}_{2}^{-}$), and hydrogen peroxide (H_2_O_2_); and lipid oxidation parameters of malondialdehyde (MDA), lipid peroxidation (LPO) were determined using commercially available kits from Nanjing Jiangcheng Bioengineering Institute (Nanjing, China; product codes were A015, A001-1, A005, A061-2, A006-1, A007-1, A003-1, A052, A018, A064-1, and A106-1, respectively) (20, 21). All measurement operation procedures strictly followed the manufacturers' protocol. Furthermore, 200 μL of final reaction solution was moved to a 96-well plate (TCP011096, JET-BIOFIL^®^, Beiden Biological Technology Co. Ltd., Nanjing, China) and was assayed via a microplate reader (Epoch, BioTek, Luzern, Switzerland).

The 2,2-diphenyl-1-picrylhydrazyl (DPPH; Pcode: 101845869, Sigma–Aldrich, St. Louis, MO, USA) scavenging activity was determined according to Tian et al. (22). Briefly, an aliquot of 0.35 mL of the milk sample was mixed with 1 mL of 0.1 mmol/L DPPH solution in a 1.5 mL tube. The mixture was centrifuged at 3,000 × g for 10 min at 4°C, and then 200 μL of supernatant was immediately transferred to a 96-well plate and incubated in the dark at room temperature for 30 min. The absorbance was analyzed at 517 nm via a microplate reader. The equation for calculation was as follows: DPPH scavenging activity (%) = (Ac – As) × 100/Ac, where Ac is the absorbance of the control and As is the absorbance of the milk sample.

### Volatile Compounds

The individual volatile compounds in milk were identified according to the method of Tian et al. (23) with a minor modification. Briefly, the gas chromatography (GC; Agilent Technologies, Santa Clara, CA, USA) conditions were: column was a fused silica capillary column (Agilent 19091S-436HP-5MS; 60 m ×250 μm ×0.25 μm); initial temperature was 40°C (kept for 2 min), warming up to 180°C (3.5°C/min), and to 310°C (10°C/min); carrier gas was He gas, and carrier gas flow rate was 1.0 mL/min; the total run time was 55 min. The MS (SCIEX-6500Qtrap; AB Allen-Bradley, Milwaukee, WI, USA) conditions were: the ion source was EI ionization, the ionization temperature was 230°C, the quadrupole temperature was 150°C, the ionization energy was 70 eV, the emission current was 34.6 μA, the multiplier voltage was 1,847 V, the interface temperature was 28°C, and the mass range was 29~500 Da. The volatile compounds were analyzed by GC/MS and run in a manual injector with 2 cm-50/30 μm DVB/CAR/PDMS StableFlex of the fiber tip. The qualitative identification of volatile compounds was noted by the standard mass spectra in NIST17 and Wiley275 libraries. The percentage of each volatile compound was obtained according to the peak area normalization method.

### Color Measurement

The color of milk sample was analyzed by using an Opto-Star equipment (Opto-Star, Matthäus, Nobitz-Klausa, Germany) according to Popov-Raljić et al. (24) with a minor modification. Briefly, a colorimeter was calibrated by a white standard plate, and then milk sample was placed in the glass cell (0.5 cm high and 2.5 cm diameter), and color values of L^*^ (lightness), a^*^ (red to green) and b^*^ (blue to yellow) were read directly at different time intervals (0, 1, 3, and 7 d). The determination was repeated three times for each of sample.

### Sensory Evaluation

The sensory evaluation of milk sample was carried out by the Institute of Animal Nutrition and Feed Science at Guizhou University. The relatively fixed full-time sensory evaluation personnel was used to form a sensory evaluation team to avoid errors caused by personnel changes in the current study. Thus, sensory evaluation of milk samples throughout product shelf life were developed by thirty panelists (including staffs, undergraduate and graduate students from Guizhou University) A total of 13 descriptive terms were selected for the major sensory attribute category as listed in Table 1 together with flavor defect and descriptions (25–27). The nine main points of the hedonic scale used are: (9) like extremely; (8) like very much; (7) like moderately; (6) like slightly; (5) neither like nor dislike; (4) dislike slightly; (3) dislike moderately; (2) dislike very much; and (1) dislike extremely. Samples were served in randomized order over panelists within each replicate. The panelists had a training session 2 d prior to evaluations to refresh their memory regarding the descriptors and the products. In these training sessions, examples of all samples that later would be evaluated were presented to the panelists.

**Table 1:** The scoring and grading of sensory evaluation of milk flavor.

**Flavor defect**	**Descriptions (reference material)**
Acid	Intensity of sour taste
Bitter	Persistent bitter taste detected at the base of the tongue
Feed	A slight barny and a feed off-flavor
Fermented/fruity	The aromatics of mildly sour and sweet associated with sauerkraut, vinegar, pineapple, apple, or other fruit
Flat	A slight intensity of oxidized off-flavor
Foreign/atypical	Various chemicals flavor, such as detergents, disinfectants, and sanitizers
Garlic/onion/weedy	Weedy, pungent odors and somewhat persistent aftertaste
Lacks freshness (stale)	Stale, chalky flavor, lack of sweetness
Malty	The malty aroma and the acid taste (or odor)
Oxidized-light (light-induced)	Burnt, burnt protein, burnt feathers, cabbage-like, and as medicinal or chemical-like off-flavors
Oxidized-metal (metal-induced)	Metallic, oily, cappy, cardboardy, stale, tallowy, painty, and fishy off-flavor
Rancid	Baby burp, feta cheese, or butyric acid aromas
Salty	Intensity of saltiness taste

### Statistical Analysis

The changes of antioxidant activity, FR, lipid peroxidation, volatile compound, and color parameters were assessed using multiple comparisons with the Duncan's test, using SAS System Version 9.1.3 (SAS Institute Inc., Cary, NC, USA). The sensory score data were analyzed using the comparison difference analysis according to Larmond (28). Each plastic tube was an experimental unit. The results were presented as the mean ± standard deviation. Pearson correlation coefficients (r) were calculated to detect the relationship between antioxidant activity and FR, lipid peroxidation, and volatile compounds in milk (29). Differences were set statistically significant at a *P*-value < 0.05.

## Results and Discussion

The temperature and pH factors that affect anthocyanin degradation could be considered to be negligible in the current research. As a result, light became the only factor impacting anthocyanin degradation in milk. Moreover, photooxidation is the main inducement of milk fat oxidation because pigments can absorb visible or ultraviolet light, resulting in photooxidation of milk lipids. Walsh et al. (30) demonstrated that photooxidation can promote the rancidity of dairy products, which was related to the wavelength, intensity and duration of light. The three components of milk can be selected as the most practical photochemically important components: (1) the proteins are related to the activated flavor of light-exposed milk; (2) the lipids are related to the other oxidized flavor; and (3) various antioxidants in milk are photoehemically changed, damaging nutritional quality (31). Reddy et al. (32) indicated that the addition of anthocyanin-rich black tea to milk could prevent oxidative damage. Hence, we found that the level of TAC in the light-protected groups was unchanged (*P* > 0.05; Table 2), suggesting that anthocyanins add antioxidant activity to milk, which could protect milk from light oxidation. This protection might be the result of single anthocyanin action and might also be related to anthocyanin synergism with other antioxidants to strengthen milk antioxidant activity (33).

**Table 2:** Effect of PCP on antioxidant capacity in milk during storage period.

**Item^**1**^**	**Storage time (d)**	**NC^**2**^**	**PC1**	**PC2**	**AC**
TAC (U/mL)	0	7.69 ± 2.19^a^	6.14 ± 2.79	7.22 ± 0.24^a^	7.01 ± 2.62
	1	5.88 ± 2.36^ab^	4.77 ± 0.63	6.87 ± 0.41^a^	6.82 ± 2.59
	3	4.89 ± 1.03^ab^	5.10 ± 1.36	4.26 ± 1.65^b^	6.50 ± 1.89
	7	2.63 ± 0.34^b^	4.18 ± 1.03	4.65 ± 1.25^b^	4.86 ± 1.94
SOD (U/mL)	0	38.03 ± 2.68^aAB^	35.00 ± 4.14^aB^	42.04 ± 4.94^A^	41.92 ± 2.06^A^
	1	24.57 ± 2.83^bC^	32.95 ± 5.44^aB^	42.93 ± 3.63^A^	43.11 ± 6.08^A^
	3	18.60 ± 7.61^bC^	25.73 ± 3.35^bB^	44.98 ± 2.15^A^	43.46 ± 2.21^A^
	7	17.12 ± 7.59^bB^	19.49 ± 5.79^bB^	43.73 ± 6.91^A^	47.28 ± 0.66^A^
GSH-Px (U)	0	60.41 ± 7.11^a^	59.56 ± 14.64^a^	71.20 ± 3.52^a^	75.78 ± 11.91^a^
	1	34.21 ± 4.40^bB^	39.38 ± 5.66^bB^	58.94 ± 8.86^abA^	64.28 ± 14.68^abA^
	3	27.57 ± 5.66^bcC^	35.41 ± 7.11^bBC^	47.92 ± 7.25^bcAB^	58.80 ± 10.99^abA^
	7	23.31 ± 5.05^cB^	26.39 ± 5.18^bB^	41.45 ± 9.65^cA^	50.41 ± 13.03^bA^
GSSG (μmol/L)	0	6.85 ± 1.96	6.57 ± 0.21	6.96 ± 0.98	7.20 ± 1.36
	1	4.72 ± 1.41	5.05 ± 1.47	6.00 ± 1.55	7.25 ± 2.59
	3	4.43 ± 1.29	4.47 ± 0.96	5.42 ± 1.10	6.56 ± 1.78
	7	4.23 ± 2.22	4.37 ± 2.09	5.08 ± 1.38	6.04 ± 0.95
GSH (μmol/L)	0	2.65 ± 1.04^a^	2.92 ± 0.51	2.28 ± 0.49	2.75 ± 0.29
	1	2.66 ± 0.36^a^	2.81 ± 1.73	2.41 ± 0.72	3.08 ± 0.77
	3	1.91 ± 0.87^ab^	2.51 ± 0.91	2.92 ± 1.34	3.08 ± 1.17
	7	0.72 ± 0.29^b^	2.92 ± 1.34	2.28 ± 1.91	2.92 ± 0.51
CAT (U/mL)	0	4.31 ± 0.77^a^	3.87 ± 0.39^a^	3.96 ± 1.09^a^	3.73 ± 0.05
	1	3.04 ± 0.59^bB^	3.79 ± 0.75^aAB^	4.69 ± 0.30^abA^	3.14 ± 0.91^B^
	3	1.70 ± 0.73^cB^	1.60 ± 0.72^bB^	3.16 ± 0.28^bA^	3.24 ± 0.54^A^
	7	1.71 ± 0.31^cB^	1.20 ± 0.48^bB^	2.80 ± 0.45^cA^	3.33 ± 0.50^A^

^1^*NC, negative control of no addition anthocyanins + exposure to fluorescent light; PC1, positive control 1 of no addition anthocyanins + protected from fluorescent light; PC2, positive control 2 of the addition of 0.3% (w/v) anthocyanins + exposure to fluorescent light; AC, the addition of 0.3% (w/v) anthocyanins + protected from fluorescent light; TAC, total antioxidant capacity; SOD, superoxide dismutase; GSH-Px, glutathione peroxidase; GSSG, oxidized glutathione; GSH, reduced glutathione; CAT, catalase; MDA, malondialdehyde*.

^2a, b^*Means with different letters within a column are significantly different between means for storage time (P < 0.05)*.

^A−D^*Means with different letters within a row are significantly different between means for milk samples (P < 0.05)*.

Milk has several kinds of enzymatic and non-enzymatic antioxidant components (SOD, GSH-Px, CAT, polyphenols, vitamin E), showing high levels of antioxidant potential and DPPH scavenging activity (34). Arreola et al. (35) did show that natural antioxidants in milk had high levels of antiproliferative activity and ${\text{O}}_{2}^{-}$ scavenging activity. However, light-exposed milk is also an exceptionally good breeding ground for bacteria, and its nutrition is destroyed during the storage period, especially decreasing antioxidative factors and natural antioxidants (36). Thus, we found that antioxidant activity in light-exposed groups tended to decrease (*P* < 0.05; Table 2) during the storage period, possibly because anthocyanins were sensitive to light, thereby decreasing milk antioxidant activity (37). This result was consistent with Gutierrez et al. (38), who indicated that TAC was significantly decreased in light-induced milk during the storage period.

Oksuz et al. (39) showed that cherry anthocyanin pigments had a relatively low level of stability in dairy food. However, the casein in milk could react with anthocyanins, leading to anthocyanins becoming stable in neutral food (40). In this study, the mean pH value was 6.52, nearly the same as that in aqueous solution, which potentially provides a condition for anthocyanin reactions with milk protein to stabilize anthocyanins in milk. Consequently, complex compounds may form between anthocyanins and proteins in milk, increasing the stability of anthocyanins to provide a necessary condition for preventing milk oxidation. Van Aardt et al. (18) showed that supplementation with 0.025% α-tocopherol in light-induced milk could prevent lipid and protein oxidation. Moreover, antioxidant enzymes can reduce reactive oxygen species and maintain the oxygen balance, thus enhancing the antioxidant activity (41, 42). In the present study, the additional anthocyanin groups showed higher (*P* < 0.05; Table 2) levels of SOD, GSH-Px, and CAT than the no additional anthocyanin groups, perhaps because PCP provided a high concentration of exogenous natural antioxidant, resulting in a greater antioxidant activity (43). These results were in accordance with Gutierrez et al. (38), who revealed that the addition of exogenous antioxidants in milk could enhance antioxidant activity. In addition, four sources of structure changed the color of different anthocyanin compositions, among which colorless colors of carbinol pseudobase and chalcone were unstable relative to the red color of flavylium cation, thereby more easily degrading to other products (44). Clifford (45) demonstrated that the structure of chalcone might degrade by oxidation reactions with high molecular weights. Light-exposed milk might be the result of changes in the four structures of anthocyanins, and they decreased in response to increasing storage days (10). Hence, antioxidant activity parameters tended to decline (*P* < 0.05; Table 2) for all groups as the storage time extended.

UFAs oxidize lipid radicals, oxidize peroxyl radicals with O_2_, and then change FA hydroperoxide with hydrogen, thus negatively affecting milk antioxidant enzymes (46). Živković et al. (47) revealed that anthocyanins might react in the water and lipid phases, which could be seen as a source of radical scavengers in milk. Moreover, Vinson et al. (48) reported that the antioxidant properties of polyphenols in milk could provide a health benefit for humans. In the current work, the AC group showed lower (*P* < 0.05; Table 3) concentrations of ${\text{O}}_{2}^{-}$ and H_2_O_2_ compared to control groups. There could be several reasons as follows: (1) the light-protected environment provided conditions for preventing milk oxidation; (2) the AC group contained high levels of anthocyanins; and (3) the H atom in anthocyanins may react with FR, leading to its stability and improved antioxidant enzymes. As expected, the AC group showed higher (*P* < 0.05) levels of DPPH scavenging activity after 1 d of storage period, providing further proof that anthocyanins commonly have reducing power and inhibition ability on oxidation of the liposome system in milk. A similar conclusion was found by Denise et al. (49), who demonstrated that adding anthocyanin-rich grape extract to milk can improve antioxidant activity by improving the DPPH radical scavenging ability.

**Table 3:** Effect of PCP on free radical parameters in milk during storage period.

**Item^**1**^**	**Storage time (d)**	**NC^**2**^**	**PC1**	**PC2**	**AC**
DPPH scavenging activity (%)	0	82.57 ± 1.68^a^	84.62 ± 0.24^a^	84.70 ± 1.19^a^	84.22 ± 0.60
	1	55.36 ± 4.53^bB^	84.86 ± 0.95^aA^	84.78 ± 0.76^aA^	85.01 ± 0.60^A^
	3	52.52 ± 2.92^bB^	83.44 ± 0.47^bA^	82.80 ± 1.19^abA^	83.48 ± 2.87^A^
	7	50.15 ± 2.49^bB^	83.28 ± 0.60^bA^	82.65 ± 0.83^bA^	84.15 ± 0.24^A^
${\text{O}}_{2}^{-}$ (U/L)	0	88.74 ± 23.45^b^	85.14 ± 4.20^b^	85.52 ± 9.98^b^	84.65 ± 12.14^ab^
	1	124.19 ± 25.06^aA^	87.29 ± 10.86^bB^	83.73 ± 8.50^bB^	79.45 ± 11.35^bB^
	3	140.80 ± 18.14^aA^	92.59 ± 4.39^bB^	97.85 ± 9.62^abB^	89.01 ± 6.38^abB^
	7	127.26 ± 19.97^a^	110.73 ± 6.61^a^	101.14 ± 9.02^a^	95.67 ± 5.08^a^
H_2_O_2_ (mmol/L)	0	66.02 ± 23.56^c^	64.80 ± 6.51^b^	69.51 ± 7.99	68.01 ± 12.11
	1	192.55 ± 29.55^bA^	80.21 ± 9.55^bB^	63.55 ± 14.41^B^	81.66 ± 13.75^B^
	3	208.75 ± 31.05^bA^	135.28 ± 13.62^aB^	82.61 ± 18.11^C^	78.91 ± 11.29^C^
	7	335.53 ± 16.24^aA^	141.71 ± 23.19^aB^	79.44 ± 12.14^C^	75.46 ± 12.19^C^

^1^*NC, negative control of no addition anthocyanins + exposure to fluorescent light; PC1, positive control 1 of no addition anthocyanins + protected from fluorescent light; PC2, positive control 2 of the addition of 0.3% (w/v) anthocyanins + exposure to fluorescent light; AC = the addition of 0.3% (w/v) anthocyanins + protected from fluorescent light. DPPH scavenging activity, 2,2-diphenyl-1-picrylhydrazyl scavenging activity; ${\text{O}}_{2}^{-}$, superoxide anion; H_2_O_2_, hydrogen peroxide*.

^2a, b^*Means with different letters within a column are significantly different between means for storage time (P < 0.05)*.

^A−D^*Means with different letters within a row are significantly different between means for milk samples (P < 0.05)*.

**Figure 1:**
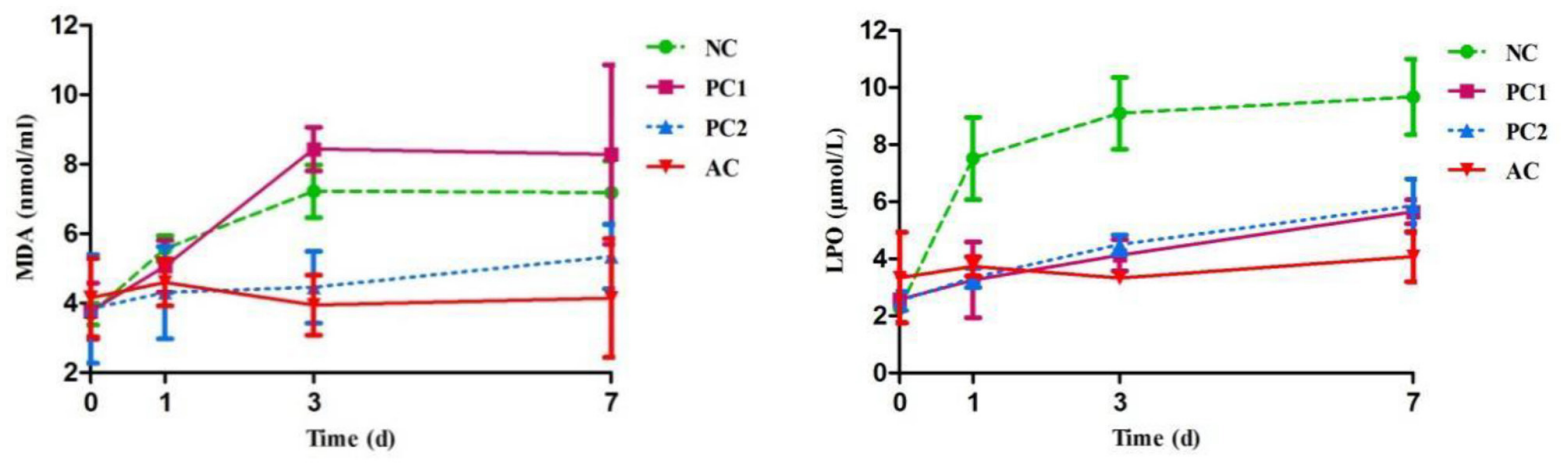
Effect of PCP on lipid peroxidation parameters in milk during storage period. NC, negative control of no addition anthocyanins + exposure to fluorescent light; PC1, positive control 1 of no addition anthocyanins + protected from fluorescent light; PC2, positive control 2 of the addition of 0.3% (w/v) anthocyanins + exposure to fluorescent light; AC, the addition of 0.3% (w/v) anthocyanins + protected from fluorescent light; MDA, malondialdehyde; LPO, lipid peroxidation.

The chemical properties of UFAs are very unstable and might appear to increase susceptibility to oxidation, producing cytotoxic lipid peroxides in milk (50). Additionally, the high reactivity of FRs have an impact on antioxidant enzymes in milk, resulting in lipid peroxidation (51). In this study, the concentrations of MDA and LPO in the control groups were increased (*P* < 0.05; Figure 1) during the storage period compared to the AC group, perhaps because light was one of the important impacting factors that increased milk lipid oxidation. This result was consistent with a previous report by Gutierrez (52), who indicated that light treatment could increase milk oxidized flavor and lipid peroxidation parameters during a refrigerated storage period. As expected, the AC group displayed the lowest (*P* < 0.05; Figure 1) MDA and LPO contents in milk at the end of the period, suggesting that PCP could inhibit lipid peroxidation and enhance antioxidative effects in light-protected milk. The reason may be that the anthocyanin-casein complex could promote autoxidation of iron, inhibiting lipid peroxidation (53). Similar to our current findings, Gualdrón et al. (54) indicated that the addition of anthocyanin-rich grape pigment could protect against oxidation of lipids and protein in yogurt during the 21-day storage period.

Twenty-two volatile compounds were determined in milk, which consisted of eight alcohols (ethanol, 1-pentanol, 1-hexanol, 1-heptanol, 1-octen-3-ol, 2-ethyl-1-hexanol, (Z)-2-octen-1-ol, and 1-octanol), three ketones (2,3-butanedione, 2-pentanone, 2,3-octanedione), five aldehydes (hexanal, heptanal, octanal, nonanal, decanal), two esters (ethyl acetate, ethyl hexanoate), and four hydrocarbons (octane, 1-nitro-hexane, dodecane, tetradecane) by GC/MS analysis (Table 4). Milk volatile compounds are produced mainly from amino acid degradation, the Maillard reaction and the thermal oxidation of lipids (55). Scanlan et al. (56) who showed that the heat processing could increase lipid oxidation in milk resulted in formation of flavor substances. Moreover, the high pasteurization could maintain milk anthocyanin level and improve the antioxidant activity (17). Anthocyanins are hydrogen donors of lipid FRs in the process of lipid oxidation and can be transformed into more stable FRs because anthocyanins can effectively intercept peroxy radicals, block chain propagation and inhibit the formation of peroxide (57). As a consequence, some volatile compounds, such as ethanol, 1-heptanol tended to increase without anthocyanin groups after heat processing compared to the anthocyanin groups. Lipid oxidation is an important cause of undesirable flavors of non-enzymatic origin in milk because O_2_ is linked to the methylene adjacent to a double bond in UFAs, thereby forming allylic hydroperoxides. Alternatively, molecular O_2_ is reduced to superoxide radicals, and it is converted into singlet O_2_ and peroxide anions. Anthocyanis can provide H atoms to peroxy radicals, thus inhibiting the oxidation of FA by chain radical termination and decreasing milk alcohol concentrations (57). As mentioned previously, light was the main factor affecting lipid oxidation, and anthocyanins can increase antioxidant activity and decrease lipid peroxidation parameters in milk. Thus, aldehydes, esters and hydrocarbons, all individual volatile compounds in the AC group remained at relatively stable (*P* > 0.05; Table 4) values during storage relative to the other three groups. Of interest, various ketones are associated with fruity and floral notes, so the presence of these volatile compounds can be considered to positively influence the flavor of milk (58). In the present research, the level of ketones increased rapidly in anthocyanin groups during storage and had higher (*P* < 0.05; Table 4) relative contents of ketones compared to the other groups, perhaps because anthocyanins could inhibit lipid oxidation, delaying the decline of milk flavor quality, and enriching the types of flavor substances (59). Conservatively, anthocyanins play a positive role in the aroma of light-protected milk, which not only enriches the taste of fruity and floral notes but also retains the nutritive value of milk, so this kind of milk might be popular with consumers.

**Table 4:** Effect of PCP on relative contents of volatile compounds in milk during storage period (%).

**Item^**1**^**	**Storage time (d)**	**NC^**2**^**	**PC1**	**PC2**	**AC**
Alcohols (8)					
Ethanol	0	4.59 ± 0.86^bA^	4.20 ± 1.10^A^	2.34 ± 0.21^B^	1.93 ± 0.17^B^
	1	5.15 ± 0.44^abA^	4.60 ± 0.26^A^	2.23 ± 0.29^B^	2.05 ± 0.34^B^
	3	5.60 ± 0.37^abA^	4.55 ± 0.29^B^	2.32 ± 0.15^C^	2.10 ± 0.23^C^
	7	5.74 ± 0.22^aA^	4.63 ± 0.31^B^	2.14 ± 0.29^C^	2.00 ± 0.32^C^
1-pentanol	0	0.43 ± 0.07^b^	0.46 ± 0.12^b^	0.46 ± 0.09^b^	0.52 ± 0.12
	1	1.94 ± 0.18^aA^	1.43 ± 0.24^aB^	0.44 ± 0.10^bC^	0.66 ± 0.21^C^
	3	2.10 ± 0.30^aA^	1.52 ± 0.33^aB^	0.52 ± 0.06^bBC^	0.69 ± 0.12^C^
	7	2.34 ± 0.33^aA^	1.42 ± 0.28^aB^	0.70 ± 0.05^aC^	0.66 ± 0.02^C^
1-hexanol	0	1.40 ± 0.15^bA^	1.50 ± 0.14^A^	1.11 ± 0.10^B^	1.06 ± 0.08^aB^
	1	2.01 ± 0.16^aA^	1.57 ± 0.18^B^	1.29 ± 0.10^C^	0.35 ± 0.05^bD^
	3	1.95 ± 0.39^aA^	1.78 ± 0.14^A^	1.16 ± 0.20^B^	0.29 ± 0.08^bcC^
	7	2.28 ± 0.30^aA^	1.80 ± 0.17^B^	1.09 ± 0.14^C^	0.21 ± 0.03^cD^
1-heptanol	0	1.39 ± 0.15^c^	1.18 ± 0.12	1.26 ± 0.14	1.18 ± 0.33
	1	2.51 ± 0.18^bA^	1.35 ± 0.05^B^	1.43 ± 0.12^B^	1.11 ± 0.12^C^
	3	2.83 ± 0.12^aA^	1.18 ± 0.17^AB^	1.410 ± 0.20^B^	0.92 ± 0.06^C^
	7	2.92 ± 0.16^aA^	1.23 ± 0.25^B^	1.31 ± 0.18^B^	0.87 ± 0.10^C^
1-octen-3-ol	0	0.11 ± 0.03^b^	0.11 ± 0.01^b^	nd	nd
	1	0.30 ± 0.06^aB^	0.13 ± 0.03^abA^	nd	nd
	3	0.36 ± 0.03^aA^	0.12 ± 0.01^abB^	nd	nd
	7	0.35 ± 0.03^aA^	0.150 ± 0.01^aB^	nd	nd
2-ethyl-1-hexanol	0	1.50 ± 0.21^b^	1.29 ± 0.08^b^	1.34 ± 0.09^c^	1.17 ± 0.25
	1	3.65 ± 0.35^aA^	2.07 ± 0.29^aB^	1.84 ± 0.08^bB^	1.34 ± 0.15^C^
	3	3.34 ± 0.50^aA^	2.20 ± 0.25^aB^	2.24 ± 0.07^aB^	1.23 ± 0.16^C^
	7	3.72 ± 0.41^aA^	2.26 ± 0.14^aB^	2.05 ± 0.14^aB^	1.18 ± 0.18^C^
(Z)-2-octen-1-ol	0	0.15 ± 0.02^b^	0.13 ± 0.02^b^	0.16 ± 0.02^ab^	0.16 ± 0.01
	1	1.58 ± 0.24^aA^	0.43 ± 0.02^aB^	0.18 ± 0.01^aC^	0.19 ± 0.01^C^
	3	1.54 ± 0.10^aA^	0.39 ± 0.04^aB^	0.16 ± 0.02^bC^	0.19 ± 0.02^C^
	7	1.50 ± 0.15^aA^	0.38 ± 0.04^aB^	0.15 ± 0.01^bC^	0.17 ± 0.02^C^
1-octanol	0	0.18 ± 0.04^bB^	0.30 ± 0.07^A^	0.25 ± 0.07^AB^	0.18 ± 0.01^aB^
	1	0.22 ± 0.05^bB^	0.32 ± 0.04^A^	0.20 ± 0.03^B^	0.11 ± 0.02^bC^
	3	0.30 ± 0.02^a^	0.26 ± 0.05	0.24 ± 0.06	nd
	7	0.32 ± 0.05^a^	0.34 ± 0.13	0.21 ± 0.05	nd
Subtotal	0	9.75 ± 0.89^bA^	9.17 ± 1.14^bA^	6.92 ± 0.26^bB^	6.20 ± 0.39^aB^
	1	17.35 ± 0.21^aA^	11.90 ± 0.30^aB^	7.60 ± 0.49^aC^	5.81 ± 0.57^abD^
	3	18.01 ± 1.17^aA^	12.00 ± 0.08^aB^	8.04 ± 0.29^aC^	5.40 ± 0.41^abD^
	7	19.16 ± 1.37^aA^	12.19 ± 0.67^aB^	7.66 ± 0.31^aC^	5.09 ± 0.48^bD^
Ketones (3)					
2,3-butanedione	0	1.68 ± 0.18	1.44 ± 0.22	1.45 ± 0.08^b^	1.46 ± 0.38^b^
	1	nd	1.46 ± 0.17^B^	2.30 ± 0.39^aA^	2.70 ± 0.15^aA^
	3	nd	1.49 ± 0.17^B^	2.31 ± 0.20^aA^	3.17 ± 0.25^aA^
	7	nd	1.51 ± 0.15^C^	2.02 ± 0.14^aB^	2.99 ± 0.15^aA^
2-pentanone	0	7.25 ± 1.05^b^	7.53 ± 0.25^b^	7.73 ± 1.15^b^	7.43 ± 0.05^b^
	1	8.33 ± 0.22^abC^	9.31 ± 0.22^aB^	16.13 ± 0.72^aA^	9.36 ± 0.42^aB^
	3	8.61 ± 0.40^aC^	9.20 ± 0.63^aBC^	18.15 ± 1.18^aA^	10.36 ± 0.57^aB^
	7	8.26 ± 0.43^abC^	9.88 ± 0.25^aBC^	17.95 ± 1.63^aA^	10.26 ± 1.03^aB^
2,3-octanedione	0	1.20 ± 0.19^b^	1.57 ± 0.26^ab^	1.62 ± 0.21^b^	1.65 ± 0.31
	1	2.41 ± 0.26^aA^	1.39 ± 0.16^bC^	2.55 ± 0.15^aA^	1.72 ± 0.12^B^
	3	2.40 ± 0.29^aA^	1.78 ± 0.11^aB^	2.92 ± 0.57^aA^	1.53 ± 0.14^B^
	7	2.60 ± 0.55^aA^	1.88 ± 0.2 1^aB^	2.95 ± 0.22^aA^	1.59 ± 0.23^B^
Subtotal	0	10.13 ± 1.09	10.54 ± 0.13^c^	10.79 ± 0.95^b^	10.54 ± 0.70^b^
	1	10.74 ± 0.05^D^	12.16 ± 0.33^bC^	20.98 ± 1.04^aA^	13.78 ± 0.28^aB^
	3	11.02 ± 0.59^C^	12.47 ± 0.82^abC^	23.38 ± 1.62^aA^	15.05 ± 0.79^aB^
	7	10.85 ± 0.97^C^	13.27 ± 0.53^aB^	22.92 ± 1.36^aA^	14.84 ± 0.87^aB^
Aldehydes (5)					
Hexanal	0	4.02 ± 0.33^bAB^	4.49 ± 0.35^cA^	4.28 ± 0.37^A^	3.60 ± 0.06^B^
	1	8.22 ± 0.78^aB^	10.18 ± 0.80^bA^	4.40 ± 0.27^C^	3.42 ± 0.30^C^
	3	8.32 ± 0.13^aB^	14.86 ± 0.98^aA^	4.48 ± 0.36^C^	3.61 ± 0.41^C^
	7	7.95 ± 0.33^aB^	16.12 ± 1.60^aA^	4.52 ± 0.30^C^	3.95 ± 0.39^C^
Heptanal	0	1.49 ± 0.17^b^	1.46 ± 0.24	1.50 ± 0.28^b^	1.44 ± 0.19
	1	2.84 ± 0.16^aA^	1.49 ± 0.17^C^	2.33 ± 0.33^aB^	1.64 ± 0.25^C^
	3	2.73 ± 0.32^aA^	1.60 ± 0.16^C^	2.13 ± 0.23^aB^	1.47 ± 0.24^C^
	7	2.93 ± 0.13^aA^	1.40 ± 0.24^C^	2.01 ± 0.24^abB^	1.43 ± 0.28^C^
Octanal	0	2.38 ± 0.03^b^	2.37 ± 0.17^b^	2.59 ± 0.27	2.30 ± 0.35
	1	5.54 ± 0.24^aA^	4.50 ± 0.25^aB^	2.35 ± 0.49^C^	2.59 ± 0.15^C^
	3	5.78 ± 0.66^aA^	4.27 ± 0.55^aB^	2.37 ± 0.22^C^	2.34 ± 0.14^C^
	7	6.03 ± 0.53^aA^	4.89 ± 0.23^aB^	2.36 ± 0.25^C^	2.24 ± 0.26^C^
Nonanal	0	38.85 ± 1.29^a^	39.21 ± 0.59^a^	38.79 ± 1.04^a^	39.67 ± 0.61
	1	18.95 ± 1.48^bC^	26.93 ± 1.73^bB^	27.35 ± 1.61^bB^	40.46 ± 1.73^A^
	3	18.72 ± 0.49^bD^	20.73 ± 1.11^cC^	27.86 ± 0.69^bB^	39.26 ± 0.87^A^
	7	19.29 ± 0.86^bC^	20.15 ± 0.46^cC^	26.67 ± 0.56^bB^	39.06 ± 0.56^A^
Decanal	0	0.32 ± 0.02^b^	0.39 ± 0.06	0.32 ± 0.02	0.32 ± 0.04
	1	0.65 ± 0.09^aA^	0.48 ± 0.06^B^	0.33 ± 0.05^C^	0.33 ± 0.04^C^
	3	0.64 ± 0.01^aA^	0.44 ± 0.07^B^	0.33 ± 0.03^C^	0.34 ± 0.03^C^
	7	0.62 ± 0.07^aA^	0.50 ± 0.06^B^	0.33 ± 0.03^C^	0.35 ± 0.03^C^
Subtotal	0	47.05 ± 1.78^a^	47.91 ± 0.82^a^	47.48 ± 0.72^a^	47.34 ± 0.50
	1	36.20 ± 0.78^bC^	43.58 ± 2.13^bB^	36.76 ± 1.30^bC^	48.44 ± 1.62^A^
	3	36.19 ± 1.29^bC^	41.90 ± 2.00^bB^	37.17 ± 0.46^bC^	47.02 ± 1.29^A^
	7	36.81 ± 0.47^bC^	43.05 ± 1.42^bB^	35.89 ± 0.37^bC^	47.03 ± 0.43^A^
Esters (2)					
Ethyl acetate	0	0.13 ± 0.03	0.12 ± 0.02	0.14 ± 0.01^a^	0.15 ± 0.02
	1	nd	nd	0.13 ± 0.00^a^	0.14 ± 0.01
	3	nd	nd	0.12 ± 0.01^bB^	0.15 ± 0.01^A^
	7	nd	nd	0.10 ± 0.01^bB^	0.14 ± 0.01^A^
Hexanoic acid, ethyl ester	0	2.10 ± 0.26^c^	2.04 ± 0.30^c^	1.78 ± 0.11	1.85 ± 0.17
	1	6.28 ± 0.72^bA^	3.07 ± 0.17^bB^	1.82 ± 0.04^C^	1.72 ± 0.14^C^
	3	8.77 ± 0.91^aA^	3.68 ± 0.37^aB^	1.92 ± 0.22^C^	1.88 ± 0.10^C^
	7	8.87 ± 0.58^aA^	3.42 ± 0.23^abB^	1.95 ± 0.05^C^	1.77 ± 0.10^C^
Subtotal	0	2.23 ± 0.27^c^	2.15 ± 0.32^c^	1.92 ± 0.11	2.00 ± 0.16
	1	6.28 ± 0.72^bA^	3.07 ± 0.17^bB^	1.95 ± 0.04^C^	1.86 ± 0.12^C^
	3	8.77 ± 0.91^aA^	3.68 ± 0.37^aB^	2.04 ± 0.22^C^	2.03 ± 0.10^C^
	7	8.87 ± 0.58^aA^	3.42 ± 0.23^abB^	2.05 ± 0.04^C^	1.91 ± 0.11^C^
Hydrocarbons (4)					
Octane	0	0.51 ± 0.15^b^	0.51 ± 0.07^c^	0.46 ± 0.07	0.44 ± 0.10
	1	1.44 ± 0.10^aA^	0.68 ± 0.06^bB^	0.46 ± 0.02^C^	0.52 ± 0.03^C^
	3	1.51 ± 0.06^aA^	0.83 ± 0.05^aB^	0.48 ± 0.05^C^	0.50 ± 0.04^C^
	7	1.57 ± 0.10^aA^	0.88 ± 0.11^aB^	0.52 ± 0.03^C^	0.52 ± 0.02^C^
1-nitro-hexane	0	1.59 ± 0.16^a^	1.54 ± 0.10^a^	1.51 ± 0.15^a^	1.53 ± 0.08
	1	0.91 ± 0.04^bB^	0.96 ± 0.10^bB^	0.95 ± 0.13^bB^	1.50 ± 0.04^A^
	3	0.67 ± 0.13^cC^	0.59 ± 0.06^cC^	0.90 ± 0.07^bB^	1.54 ± 0.11^A^
	7	0.66 ± 0.05^cC^	0.62 ± 0.05^cC^	0.90 ± 0.08^bB^	1.60 ± 0.06^A^
Dodecane	0	0.30 ± 0.06^b^	0.27 ± 0.04^b^	0.35 ± 0.06^a^	0.29 ± 0.05
	1	0.99 ± 0.12^aA^	0.52 ± 0.12^aB^	0.26 ± 0.05^bC^	0.22 ± 0.02^C^
	3	0.87 ± 0.09^aA^	0.60 ± 0.05^aB^	0.24 ± 0.04^bC^	0.28 ± 0.03^C^
	7	0.86 ± 0.12^aA^	0.62 ± 0.04^aB^	0.27 ± 0.01^bC^	0.27 ± 0.05^C^
Tetradecane	0	1.34 ± 0.11^a^	1.42 ± 0.10^b^	1.43 ± 0.12	1.17 ± 0.29
	1	1.01 ± 0.16^bC^	1.65 ± 0.10^aA^	1.26 ± 0.16^B^	1.33 ± 0.11^B^
	3	0.83 ± 0.06^bC^	1.70 ± 0.15^aA^	1.25 ± 0.20^B^	1.43 ± 0.22^AB^
	7	0.87 ± 0.11^bC^	1.78 ± 0.08^aA^	1.13 ± 0.11^B^	1.33 ± 0.11^B^
Subtotal	0	3.74 ± 0.04^b^	3.74 ± 0.27	3.76 ± 0.17^a^	3.43 ± 0.22
	1	4.34 ± 0.36^aA^	3.81 ± 0.13^B^	2.93 ± 0.19^bC^	3.57 ± 0.18^B^
	3	3.88 ± 0.08^bA^	3.72 ± 0.28^A^	2.86 ± 0.12^bB^	3.76 ± 0.22^A^
	7	3.96 ± 0.05^bA^	3.89 ± 0.15^A^	2.82 ± 0.21^bB^	3.72 ± 0.21^A^

^1^*NC, negative control of no addition anthocyanins + exposure to fluorescent light; PC1, positive control 1 of no addition anthocyanins + protected from fluorescent light; PC2, positive control 2 of the addition of 0.3% (w/v) anthocyanins + exposure to fluorescent light; AC, the addition of 0.3% (w/v) anthocyanins + protected from fluorescent light*.

^2a, b^*Means with different letters within a column are significantly different between means for storage time (P < 0.05)*.

^A−D^*Means with different letters within a row are significantly different between means for milk samples (P < 0.05)*.

Anthocyanins have the ability to scavenge oxygen FR in milk and are the main contributors to antioxidant potential in milk (60). Thus, stronger correlations (*P* < 0.05; Table 5) were noted between some antioxidant activities and DPPH scavenging activity as well as total ketones. These results were in agreement with Mann et al. (61), who demonstrated that there was a stronger correlation between TAC and DPPH scavenging activity in milk. Moreover, numerous FRs and lipid peroxidations are produced in the process of lipid oxidation (62, 63). Polyphenols could protect milk oxidation by removing the concentrations of FRs and reducing lipid peroxidation, leading to the maintenance of volatile compounds in milk (64). Therefore, the correlation analysis showed negative (*P* < 0.05; Table 5) correlations between antioxidant activity and ${\text{O}}_{2}^{-}$, H_2_O_2_, MDA, LPO, alcohols, and esters in milk. Consistent with our observations, Stapelfeldt et al. (65) indicated that there were negative correlations between FR concentrations and antioxidant levels in milk.

**Table 5:** Pearson correlation coefficients between antioxidant activity, free radical, lipid peroxidation, and volatile compounds in milk.

**Item^**a**^**		**DPPH**	** ${\text{O}}_{2}^{-}$ **	**H_**2**_O_**2**_**	**MDA**	**LPO**	**Alcohols**	**Ketones**	**Aldehydes**	**Esters**	**Hydrocarbons**
TAC	r	−0.0411	−0.1542	−0.1341	−0.4537	−0.2005	0.0420	−0.5060	0.3012	0.0357	0.2243
	P	0.8800	0.5686	0.6206	0.0775	0.4564	0.8773	0.0455	0.2570	0.8955	0.4037
SOD	r	0.6970	−0.7615	−0.8230	−0.8287	−0.7142	−0.9227	0.4996	0.4128	−0.8466	−0.6261
	P	0.0027	0.0006	<0.0001	<0.0001	0.0019	<0.0001	0.0488	0.1121	<0.0001	0.0095
GSH-Px	r	0.6195	−0.8021	−0.7508	−0.8312	0.0619	−0.8035	0.0028	0.6719	−0.7387	−0.3196
	P	0.0105	0.0002	0.0008	<0.0001	0.8134	0.0002	0.9919	0.0044	0.0011	0.2276
GSSG	r	0.5612	−0.7616	−0.7017	−0.8408	−0.7582	−0.7771	−0.0835	0.7553	−0.6945	−0.2097
	P	0.0237	0.0006	0.0024	<0.0001	0.0007	0.0004	0.7586	0.0007	0.0028	0.4357
GSH	r	0.8133	−0.5997	−0.7936	−0.4185	−0.6945	−0.6517	0.1501	0.5373	−0.7432	−0.1274
	P	0.0001	0.0141	0.0002	0.1066	0.0028	0.0062	0.5789	0.0318	0.0010	0.6382
CAT	r	0.4469	−0.6722	−0.6473	−0.8786	−0.7119	−0.5162	0.0371	0.3801	−0.5692	−0.2454
	P	0.0827	0.0043	0.0067	<0.0001	0.0020	0.0407	0.8914	0.1465	0.0214	0.3597

^a^*TAC, total antioxidant capacity; SOD, superoxide dismutase; GSH-Px, glutathione peroxidase; GSSG, oxidized glutathione; GSH, reduced glutathione; CAT, catalase; DPPH, 2,2-diphenyl-1-picrylhydrazyl scavenging activity; ${\text{O}}_{2}^{-}$ superoxide anion; H_2_O_2_, hydrogen peroxide; MDA, malondialdehyde; LPO, lipid peroxidation*.

Anthocyanins have been associated with the color change of milk added with purple corn extract; and the color values were impacted on the anthocyanin concentration, storage temperature and storage duration (66). All of milk samples were pasteurized and placed in tubes, and kept at 4°C during the 7 d period in the present research. A previous study has been showed that adding of anthocyanin in milk can be stored at 4°C temperature condition, and the degrade rate of anthocyanins should be extended (67). Therefore, adding of anthocyanin did not impact (*P* > 0.05; Table 6) on the color values of L^*^ and b^*^ in milk among all groups during the entire storage period. However, light is one of the major environmental factors that affecting anthocyanin degradation (68). As a result, anthocyanin decreased significantly in response to increasing storage time (11). More specifically, secondary complexes may also form between polyphenols and proteins, which can reduce the color rendering ability of the anthocyanins. These previous reports lead us to presume that PCP could keep the stability of anthocyanin concentration in light-protected milk. Accordingly, the red color of milk began to decrease (*P* < 0.05) in PC2 group at 1 d storage, but it did not differ (*P* > 0.05) in the light-protected group during the whole storage period. In summary, our results suggest that adding of PCP in light-protected milk can maintain their color value at a low temperature condition. Consistent with our result, Sawale et al. (69) who found that addition of anthocyanin-rich *Pueraria tuberosa* extract to milk can decrease lightness value, whereas it can increase yellowness and redness values.

**Table 6:** Effect of PCP on color parameters in milk during storage period.

**Item^**1**^**	**Storage time (d)**	**NC^**2**^**	**PC1**	**PC2**	**AC**
L*	0	77.25 ± 0.87	77.83 ± 1.99	74.33 ± 2.08	73.23 ± 0.63
	1	78.88 ± 1.15	78.45 ± 2.18	75.88 ± 1.28	73.55 ± 0.65
	3	77.75 ± 2.94	78.25 ± 0.40	73.73 ± 2.01	73.75 ± 3.08
	7	77.68 ± 1.77	77.50 ± 2.11	74.70 ± 3.11	73.63 ± 3.76
a*	0	−2.88 ± 0.35	−2.80 ± 0.73	−1.68 ± 0.25^a^	−1.80 ± 0.43
	1	−2.78 ± 0.68	−2.85 ± 0.52	−2.13 ± 0.28^ab^	−2.10 ± 0.26
	3	−2.70 ± 0.50	−2.93 ± 0.54	−2.28 ± 0.36^b^	−2.13 ± 0.41
	7	−2.73 ± 0.46	−3.08 ± 0.43	−2.35 ± 0.29^b^	−2.05 ± 0.42
b*	0	4.08 ± 0.54	4.20 ± 0.43	6.38 ± 0.93	6.25 ± 0.65
	1	4.25 ± 0.82	4.40 ± 0.71	6.65 ± 1.33	6.38 ± 0.59
	3	4.20 ± 0.50	4.33 ± 0.50	6.13 ± 0.94	6.45 ± 0.89
	7	4.58 ± 0.75	4.20 ± 0.71	6.40 ± 0.67	6.43 ± 0.64

^1^*NC, negative control of no addition anthocyanins + exposure to fluorescent light; PC1, positive control 1 of no addition anthocyanins + protected from fluorescent light; PC2, positive control 2 of the addition of 0.3% (w/v) anthocyanins + exposure to fluorescent light; AC, the addition of 0.3% (w/v) anthocyanins + protected from fluorescent light*.

^2a, b^*Means with different letters within a column are significantly different between means for storage time (P < 0.05)*.

In consideration of consumer acceptance, enrichment of pasteurized milk with natural antioxidants without compromising the sensory attributes of milk is very essential (70). Furthermore, sensory evaluation is necessary during the development of the dairy products. The attributes of acid, bitter, feed, fermented/fruity, flat, foreign/atypical, garlic/onion/weedy, lacks freshness (stale), malty, oxidized-light (light-induced), oxidized-metal (metal-induced), rancid and salty for the milk samples were evaluated as being in the range of 5 to 9, suggesting that adding of anthocyanin in milk had no negative impact on the sensory evaluation of consumers. Of interest, adding of anthocyanins in milk may have bitter taste because attributed to the interaction of polyphenol-salivary protein (69). However, the lipid oxidation can decrease nutritive value and sensory quality in milk (59). Thus, adding of 0.3% PCP had the potential to improve the acceptability of consumers. These results showed that no attributes changed if adding of PCP in the pasteurized milk. A previous study has shown that purple corn anthocyanins can remain stable at 80°C (71). It was safe to assume that adding of PCP in pasteurization of fresh milk could maintain the active constituent anthocyanin content in dark condition and improve consumer health. In the present study, most of sensory evaluation parameters in anthocyanin groups showed relatively (Table 7) higher values compared to without anthocyanin groups, perhaps due to milk supplementation of PCP and light prevention during storage period. Current experiment results are in agreement with the finding of Ma et al. (67), who observed adding of anthocyanins in milk had no negative effect on the sensory quality parameters. Similarly, Sawale et al. (69) who demonstrated that no adverse effect on sensory attributes of milk with 0.4% anthocyanin-rich *Pueraria tuberosa* extract. Overall, these results indicated that the process of anthocyanin and light prevention did not influence the sensory characteristics of cow milk; most importantly, adding of PCP might not affect the consumer acceptance of products containing pasteurized milk as a major ingredient. Therefore, from the view of practical application, adding of anthocyanin in pasteurized milk was a feasible and effective method.

**Table 7:** Effect of PCP on sensory scores in milk during storage period.

**Sensory attributes^**1**^**	**Storage time (d)**	**NC^**2**^**	**PC1**	**PC2**	**AC**
Acid	0	8.10 ± 0.36	7.90 ± 0.66	7.93 ± 0.32	7.97 ± 0.39
	1	7.93 ± 0.42	7.93 ± 0.12	7.97 ± 0.21	7.73 ± 0.41
	3	7.57 ± 0.25	7.70 ± 0.75	7.70 ± 0.36	7.83 ± 0.49
	7	7.73 ± 0.38	7.67 ± 0.15	7.70 ± 0.17	7.80 ± 0.20
Bitter	0	7.60 ± 0.60	7.63 ± 0.32	7.53 ± 0.76	7.43 ± 0.40
	1	7.63 ± 0.65	7.50 ± 0.46	7.60 ± 0.53	7.50 ± 0.46
	3	7.30 ± 0.49	7.60 ± 0.61	7.40 ± 0.53	7.37 ± 0.32
	7	7.53 ± 0.50	7.43 ± 0.40	7.33 ± 0.29	7.47 ± 0.50
Feed	0	7.50 ± 0.41	7.57 ± 0.40	7.63 ± 0.65	7.70 ± 0.26
	1	7.10 ± 0.46	7.17 ± 0.19	7.13 ± 0.31	7.30 ± 0.44
	3	7.27 ± 0.31	7.37 ± 0.32	6.97 ± 0.35	7.13 ± 0.32
	7	7.07 ± 0.72	7.00 ± 0.20	7.03 ± 0.25	7.33 ± 0.31
Fermented/fruity	0	8.07 ± 0.21^a^	8.13 ± 0.31^a^	7.97 ± 0.21^a^	8.20 ± 0.26^a^
	1	7.27 ± 0.31^ab^	7.50 ± 0.27^b^	7.37 ± 0.55^ab^	7.57 ± 0.25^b^
	3	6.87 ± 0.24^bc^	7.10 ± 0.24^bc^	7.00 ± 0.44^bc^	7.13 ± 0.47^bc^
	7	6.40 ± 0.40^c^	6.83 ± 0.32^c^	6.53 ± 0.32^c^	6.93 ± 0.46^c^
Flat	0	7.33 ± 0.31^a^	7.40 ± 0.35^a^	7.67 ± 0.31^a^	7.53 ± 0.22^a^
	1	6.60 ± 0.53^b^	6.43 ± 0.29^b^	7.07 ± 0.21^b^	7.07 ± 0.33^b^
	3	6.43 ± 0.35^bB^	6.97 ± 0.25^abA^	7.10 ± 0.26^bA^	7.23 ± 0.21^bA^
	7	6.30 ± 0.26^bC^	6.40 ± 0.36^bBC^	6.87 ± 0.31^bAB^	7.33 ± 0.24^abA^
Foreign/atypical	0	8.17 ± 0.15^a^	8.03 ± 0.25	8.27 ± 0.18^a^	8.20 ± 0.26^a^
	1	7.80 ± 0.20^ab^	7.90 ± 0.50	7.70 ± 0.22^b^	7.53 ± 0.23^b^
	3	7.67 ± 0.35^b^	7.77 ± 0.31	7.57 ± 0.38^b^	7.33 ± 0.32^b^
	7	7.63 ± 0.25^b^	7.50 ± 0.44	7.63 ± 0.25^b^	7.68 ± 0.25^b^
Garlic/onion/weedy	0	7.63 ± 0.21^a^	7.50 ± 0.26^a^	7.47 ± 0.42	7.63 ± 0.21
	1	7.13 ± 0.24^bc^	6.93 ± 0.22^b^	7.27 ± 0.45	7.17 ± 0.40
	3	7.20 ± 0.20^bA^	6.40 ± 0.36^cB^	7.13 ± 0.23^A^	7.17 ± 0.29^A^
	7	6.90 ± 0.17^cB^	6.77 ± 0.25^bcB^	7.30 ± 0.33^A^	7.07 ± 0.31^AB^
Lacks freshness (stale)	0	7.30 ± 0.26^a^	7.07 ± 0.27^a^	7.27 ± 0.25^a^	7.13 ± 0.27^a^
	1	5.87 ± 0.21^b^	6.43 ± 0.29^b^	6.53 ± 0.46^b^	6.27 ± 0.46^b^
	3	6.03 ± 0.24^b^	6.17 ± 0.24^b^	6.13 ± 0.42^b^	5.93 ± 0.45^b^
	7	5.93 ± 0.29^b^	5.97 ± 0.55^b^	6.03 ± 0.56^b^	6.30 ± 0.26^b^
Malty	0	7.23 ± 0.25	7.07 ± 0.70	7.13 ± 0.27	7.20 ± 0.20
	1	6.73 ± 0.42	7.18 ± 0.29	6.87 ± 0.31	6.93 ± 0.35
	3	7.03 ± 0.30	6.63 ± 0.25	6.77 ± 0.25	7.03 ± 0.42
	7	6.77 ± 0.45	6.87 ± 0.45	6.80 ± 0.52	6.87 ± 0.45
Oxidized-light (light-induced)	0	8.07 ± 0.31^a^	7.97 ± 0.45	7.93 ± 0.60	8.07 ± 0.23
	1	7.60 ± 0.20^abBC^	7.83 ± 0.21^AB^	7.47 ± 0.22^C^	8.03 ± 0.21^A^
	3	7.23 ± 0.21^bB^	7.33 ± 0.31^B^	7.57 ± 0.25^B^	8.20 ± 0.46^A^
	7	6.07 ± 0.47^cB^	7.40 ± 0.50^A^	7.43 ± 0.25^A^	7.93 ± 0.15^A^
Oxidized-metal (metal-induced)	0	7.60 ± 0.20^a^	7.47 ± 0.50^a^	7.50 ± 0.36^a^	7.37 ± 0.23^a^
	1	6.83 ± 0.47^ab^	7.23 ± 0.38^ab^	7.00 ± 0.60^b^	6.77 ± 0.23^b^
	3	5.90 ± 0.61^cB^	6.73 ± 0.29^bA^	6.57 ± 0.25^bAB^	6.87 ± 0.38^abA^
	7	6.17 ± 0.35^bB^	7.20 ± 0.20^abA^	6.67 ± 0.25^bAB^	7.10 ± 0.36^abA^
Rancid	0	8.03 ± 0.25^a^	7.93 ± 0.45^a^	7.80 ± 0.20	8.07 ± 0.42
	1	6.80 ± 0.26^bB^	7.23 ± 0.21^bA^	7.37 ± 0.55^A^	7.77 ± 0.22^A^
	3	7.00 ± 0.20^bB^	7.40 ± 0.40^abAB^	7.40 ± 0.40^AB^	7.90 ± 0.26^A^
	7	6.67 ± 0.25^bC^	7.20 ± 0.20^bB^	7.50 ± 0.30^AB^	7.70 ± 0.24^A^
Salty	0	6.87 ± 0.43	6.87 ± 0.47	6.77 ± 0.25	6.93 ± 0.25
	1	6.53 ± 0.62	6.67 ± 0.47	6.70 ± 0.66	7.07 ± 0.31
	3	6.67 ± 0.25	6.50 ± 0.44	6.77 ± 0.68	7.03 ± 0.23
	7	6.57 ± 0.43	6.53 ± 0.50	6.53 ± 0.50	6.70 ± 0.30

^1^*The sensory attributes are identified in Table 1*.

^2a, b^*Means with different letters within a column are significantly different between means for storage time (P < 0.05)*.

^A−D^*Means with different letters within a row are significantly different between means for milk samples (P < 0.05)*.

## Conclusions

The present results indicated that the addition of PCP had the potential to prevent milk oxidation because: (1) it could enhance antioxidant activity and reduce the extent of FRs and lipid peroxidation parameters, (2) it could maintain volatile compounds and enrich the taste of fruity and floral notes, and (3) it could increase the sensory scores to improve the acceptability of consumers in light-protected milk within the 7-day storage period. Further studies are needed to elucidate the mechanism by which anthocyanins protect against milk oxidation.

## Data Availability Statement

The original contributions presented in the study are included in the article, further inquiries can be directed to the corresponding author.

## Ethics Statement

The animal study was reviewed and approved by the Rules of the College of Animal Science, Guizhou University.

## Author Contributions

X-ZT conducted all the experiments, data analyzing, and writing—original draft preparation and project administration. XW, CB, Q-YL, and J-XL contributed by the investigation, data curation. QL conducted methodology, supervising data, and project administration. All authors contributed to the article and approved the submitted version.

## Funding

This work was funded by the Science and Technology Project of Guizhou Province (Qiankehe foundation-ZK[2021] General 164), Youth Science and Technology Talent Development Project of Guizhou Province (Qianjiaohe KY [2022]150), the Cultivating Project of Guizhou University (2019-33), and the Start-up Funds of Guizhou University (2016-76; 2019-26), respectively.

## Conflict of Interest

The authors declare that the research was conducted in the absence of any commercial or financial relationships that could be construed as a potential conflict of interest.

## Publisher's Note

All claims expressed in this article are solely those of the authors and do not necessarily represent those of their affiliated organizations, or those of the publisher, the editors and the reviewers. Any product that may be evaluated in this article, or claim that may be made by its manufacturer, is not guaranteed or endorsed by the publisher.
